# Simultaneous Compositional and Grain Size Measurements Using Laser Opto-Ultrasonic Dual Detection for Additive Manufacturing

**DOI:** 10.3390/ma13102404

**Published:** 2020-05-23

**Authors:** Yuyang Ma, Xiujuan Hu, Zhenlin Hu, Ziqian Sheng, Shixiang Ma, Yanwu Chu, Qing Wan, Wei Luo, Lianbo Guo

**Affiliations:** 1 Wuhan National Laboratory for Optoelectronics (WNLO), Huazhong University of Science and Technology, Wuhan 430074, China; yyma@hust.edu.cn (Y.M.); huzhenlin@hust.edu.cn (Z.H.); m201872800@hust.edu.cn (Z.S.); shixiangma@hust.edu.cn (S.M.); wikiman@hust.edu.cn (Y.C.); m201772785@hust.edu.cn (Q.W.); 2 School of Physics, Huazhong University of Science and Technology, Wuhan 430074, China; BubbleXiu@hust.edu.cn; 3 School of Optical and Electronic Information, Huazhong University of Science and Technology, Wuhan 430074, China; luowei@hust.edu.cn

**Keywords:** elemental composition, grain size distribution, wire + arc additive manufacturing, LOUD detection

## Abstract

Metal-based additive manufacturing (AM) is a disruptive technique with great potential across multiple industries; however, its manufacturing quality is unstable, leading to an urgent requirement for component properties detection. The distribution of grain size has an important effect on many mechanical properties in AM, while the distribution of added elements, such as titanium (Ti), has a measurable effect on the grain size of an aluminum (Al) alloy. Therefore, the detection of the distributions of grain size and elements is of great significance for AM. In this study, we investigated the distribution of grain size and elements simultaneously for wire + arc additive manufacturing (WAAM) with an Al alloy using laser opto-ultrasonic dual (LOUD) detection. The average grain size obtained from the acoustic attenuation of ultrasonic signals was consistent with the results of electron backscatter diffraction (EBSD), with a coefficient of determination (R^2^) of 0.981 for linear fitting. The Ti element distribution obtained from optical spectra showed that the enrichment of Ti corresponded to the grain refinement area in the detected area. The X-ray diffraction (XRD) spectra showed that the spectral peaks were moved from Al to AlTi and Al_2_Ti forms in the Ti-rich areas, which confirmed the LOUD results. The results indicated that LOUD detection holds promise for becoming an effective method of analyzing the mechanical and chemical properties of components simultaneously, which could help explain the complex physical and chemical changes in AM and ultimately improve the manufacturing quality.

## 1. Introduction

As a disruptive technique with great potential across multiple industries, additive manufacturing (AM) has been developed in the energy, aerospace, and biomedical industries [1]. However, its manufacturing quality is unstable with fluctuant parameters, leading to an urgent requirement for the detection of component properties. The distribution of grain size in components has been investigated for its connection to porosity [2] as well as most mechanical properties, such as stress, hardness, and tensile strength [3,4,5,6,7]. Therefore, characterization of the distribution of grain size is of great significance [8]. The methods of grain size detection are mainly divided into two types: destructive and nondestructive. Destructive detection methods, such as optical metallography and electron backscatter diffraction (EBSD) [9,10], are limited in engineering. Their shortcomings include the fact that they are time consuming, that they involve microdetection, and that they result in irreversible damage to the material. Therefore, nondestructive detection, including the eddy current method [11], the magnetic Barkhausen noise technique [12], terahertz technology [13], infrared thermography [13], and ultrasonic detection [14], are widely used in the detection of large areas and in-service material for engineering.

With the advantages of high detection depth, easy automation, applicability to almost all metals, and safe use, ultrasonic detection has been widely used in the grain size measurement of polycrystalline metallic materials. For example, Bai et al. [14] studied the grain size distribution in a titanium (Ti) alloy using laser-generated ultrasound, with the results showing that the attenuation of laser-generated ultrasound could characterize the grain size. Bouda et al. [15] compared the ultrasonic velocities method with the ultrasonic attenuation method for grain size measurement in steel. Li et al. [16] reported an evaluation model based on the multiscale ultrasonic attenuation coefficient to detect the grain size in 304 stainless steel. 

One of the most significant reasons that grain size differs in materials is the distribution of added elements. Tamura et al. [17] indicated that added elements, such as iron (Fe), manganese (Mn), beryllium (Be), and zirconium (Zr), had a grain coarsening effect on the magnesium (Mg)–9% aluminum (Al) alloy. Sigworth et al. [2] reported that the Ti content made a difference to the grain size distribution in aluminum–silicon (Al–Si), aluminum–copper (Al–Cu), aluminum–magnesium (Al–Mg), and aluminum–zinc (Al–Zn) alloys. However, there is no method that can simultaneously analyze the large distribution of grain size and the elements. Therefore, a macroscopic, simultaneous, and visualized detection method for the distribution of grain size and elements is urgently needed. Due to the great detection of elemental information, structural defects, and residual stress using laser opto-ultrasonic dual (LOUD) detection in wire + arc additive manufacturing (WAAM) [18], the detection of and relationship between the grain size and elements were simultaneously studied in this paper with the LOUD method. The optical emission and ultrasonic waves were generated when the laser ablated the samples. The distribution of elements was obtained from the optical spectra, while the distribution of grain size was detected via the ultrasonic wave signals.

## 2. Materials and Methods

### 2.1. Experimental Setup and Materials 

**Figure 1:**
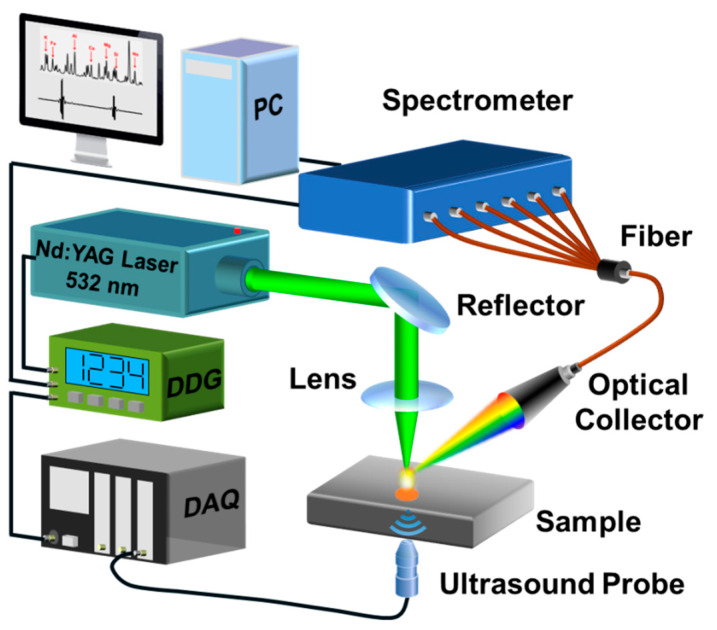
Schematic diagram of the experimental setup. A 532 nm Nd: YAG laser was used to ablate the sample surface. The optical spectrum signals were collected by an optical collector and guided by a fiber to a spectrometer. The ultrasonic signals were gathered with an ultrasound probe and guided to a data acquisition card (DAQ). A digital delay generator (DDG) was utilized to trigger the laser, DAQ card, and charge-coupled device (CCD) detector in the experiments.

A schematic diagram of the LOUD setup is shown in Figure 1. The laser beam was generated by a 532 nm Q-switched Nd: YAG laser (Nimma-400, Beamtech Optronics Co., Ltd., Beijing, China), and it was reflected and then focused onto the surface of the wire + arc additive manufacturing (WAAM) Al alloy sample by a focal lens (f = 150mm). The experiment was performed in the open air. The plasma emission was gathered with an optical collector and then guided by an optical fiber to a six-channel, charge-coupled device (CCD) spectrometer (AvaSpec-ULS4096CL-EVO, Avantes B.V., Apeldoorn, Netherlands) which covered an overall range of 200–313, 310–400, 397–473, 470–593, 590–693, and 690–800 nm. The integration time delay was set to 2 μs to obtain high spectral intensity and signal-to-background ratio (SBR). Each spectrum was accumulated from ten shots. Meanwhile, a 20 MHz ultrasonic probe (V316 SU, Olympus Co., Tokyo, Japan) coupled to the surface of the WAAM Al alloy sample was used to detect the ultrasonic signals. A data acquisition card (NI 5160, National Instruments Co., Austin, TX, USA) with a sampling frequency of 2 GHz was used to record the ultrasonic signals. A digital delay generator (LDG 3.0, Wuhan N&D Laser Engineering Co., Ltd., Wuhan, China) was utilized to trigger the laser, CCD detector, and data acquisition card (DAQ) in the experiments.

**Figure 2:**
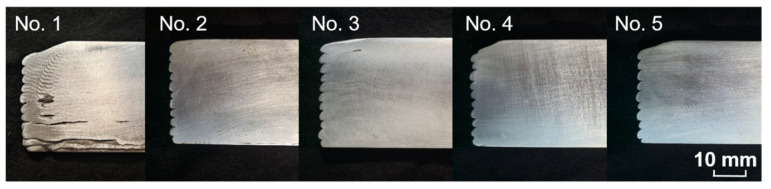
Pictures of five wire + arc additive manufacturing (WAAM) samples with different processing parameters.

WAAM technology, which has attracted significant interest due to its advantages of high deposition efficiency, high material utilization, and low equipment costs, was used in the experiment [19]. As shown in Figure 2**,** five WAAM single-wall samples were manufactured under the typical variable polarity cold metal transfer (VP CMT) mode, and the processing parameters are shown in Table 1. The feedstock wire was ER2319 Al alloy filler (the chemical compositions of which are given in Table 2) with a diameter of 1.2 mm. A 10 mm-thick pure Al plate was used as the forming substrate. The shielding gas was 99.9% argon with a flow rate of 25 L/min. The manufacturing setup was comprised of a Fronius CMT-Advanced 4000 power source, a Fronius VR 7000 wire feeder, and a FANUC M-710iC/50 robot (Fronius International GmbH, Wels, Austria). 

### 2.2. Laser Opto-Ultrasonic Dual (LOUD) Detection

#### 2.2.1. Generation of an Opto-Ultrasonic Signal

**Figure 3:**
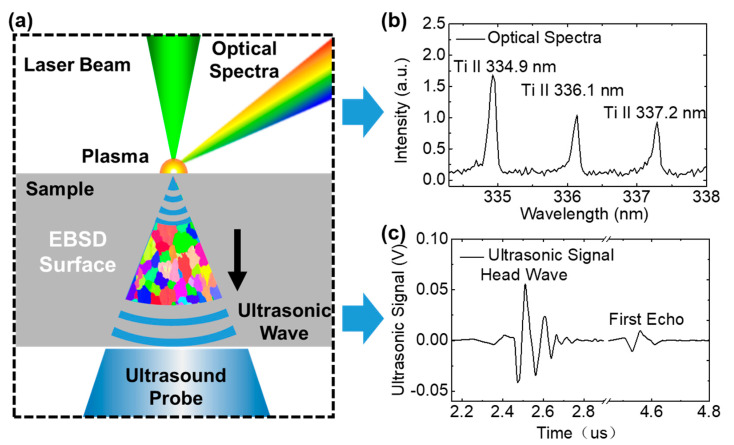
Schematic of optical spectra and ultrasonic signals simultaneously detected using laser opto-ultrasonic dual (LOUD) detection. (**a**) The optical spectrum and ultrasonic wave generated by laser ablation. The electron backscatter diffraction (EBSD) surface was parallel to the ultrasonic wave propagation direction. (**b**) Optical spectra peaks of Ti collected from the samples. (**c**) The ultrasonic signals show the amplitude attenuation from head wave to first echo.

As shown in Figure 3a, when the laser pulse energy was higher than the ablation threshold of the materials, the surface was ablated and vaporized to generate plasma. The optical spectra, which could be used to analyze the elements, were generated by the transition of electrons in the plasma. At the same time, the ultrasonic wave transmitted inside the sample was generated by both the material ablation and plasma expansion.

#### 2.2.2. Optical Spectral Signal Processing of Elements

Due to the different energy levels and absorbed energy of electron transition in the atoms, the wavelengths of the emission lines from different elements are different, and these can be used to analyze the types of elements [20,21,22]. As shown in Figure 3b, there were three emission lines for Ti, including the spectra of Ti II 334.9, 336.1, and 337.2 nm. Titanium II 334.9 nm was selected as the analytical line because the intensity was higher than other lines. Under certain conditions, such as the same matrix, the optical spectra intensity is proportional to the elemental content. For correcting the self-absorption effect in the Al alloy WAAM sample, based on previous works, the relationship can be expressed as [23,24,25]

(1) $$I\left(C\right)=A\left(1-e^{-\alpha C}\right)+I_{b},$$

where I(C) is the emission spectral line intensity, C is the element concentration, A represents the relative emission spectral line intensity, α is the factor of spectral self-absorption, and $I_{b}$ is the spectral background intensity. Therefore, the distribution of element concentration could be reflected as the distribution of spectral intensity.

#### 2.2.3. Ultrasonic Signal Processing of Grain Size 

As shown in Figure 3a, the laser beam ablation of the sample excitation surface excited a vertical propagated ultrasonic longitudinal wave, which could be detected by the ultrasonic probe on the receiving surface. A typical laser ultrasonic signal measured from Sample 1 is shown in Figure 3c. The head wave signal observed at about 2.4 μs corresponded to the initial wave which was transmitted to the receiving surface first. Then, the first echo signal was observed at about 4.5 μs, which corresponded to the initial wave having transmitted to the ultrasonic probe on the receiving surface for the second time. It was found that the amplitude of the ultrasonic signal had been attenuated. According to the ratio of ultrasonic wavelength to grain size, the scattering effect of the laser ultrasonic wave and the grain size in this study was stochastic scattering [26,27], which showed a linear relationship,

(2) $$\alpha =CDf^{2},$$

where α is the acoustic attenuation, C is the elastic constant depending on the elastic stiffness and anisotropy of the single crystal, D is the grain size, and f is the ultrasonic wave frequency. The acoustic attenuation can be expressed by the ratio of the signal amplitude of the head wave and the echo. Therefore, given the propagation distance, the acoustic attenuation is related to the grain size of the materials, which can be expressed as [15]

(3) $$\alpha =\frac{1}{2d}20\mathrm{lg}\left(\frac{A_{h}}{A_{e}}\right),$$

where α is the acoustic attenuation coefficient, d represents the thickness of the sample in the direction of acoustic wave propagation, $A_{h}$ is the amplitude of the head wave, and $A_{e}$ is the amplitude of the first echo.

As shown in Equations (2) and (3), the acoustic attenuation coefficient${\;\alpha }_{c},\;$that is used to characterize the grain size, can be obtained by measuring the head wave and first echo [14].

## 3. Results and Discussion

### 3.1. Grain Size Measurements of LOUD and EBSD

**Figure 4:**
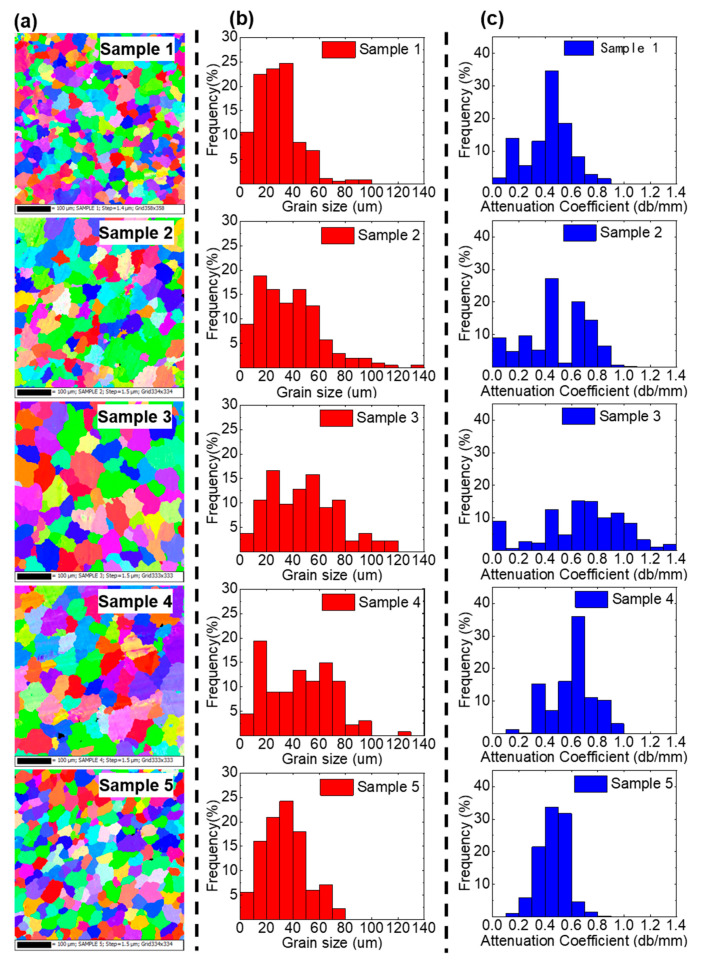
The measurements of grain size using EBSD and LOUD. (**a**) The EBSD maps of Samples 1–5 showing the grain size distribution. (**b**) The grain size measurements from the EBSD maps. (**c**) The distributions of grain size determined from the acoustic attenuation coefficient by LOUD for Samples 1–5, presented as blue histograms.

The surface of each sample was milled flat and then cleaned with alcohol. To avoid unstable manufacturing on the edge area of the sample, the central areas of Samples 1–5 were selected for detection. The same areas were used for detection by EBSD and LOUD. As shown in Figure 3a, the EBSD surface was parallel to the ultrasonic wave propagation direction. As shown in Figure 4a, the EBSD maps were carried out by destructive metallographic observation (GeminiSEM 300 TSL (EDAX), Carl Zeiss Inc. Co., Oberkochen, Germany). The measured data of the grain size distribution determined from the EBSD maps were plotted as red histograms in Figure 4b, which shows the proportion of grains with different sizes in the total grains. Then, based on Equation 3, the measured data of the acoustic attenuation coefficient from LOUD detection were plotted as blue histograms in Figure 4c, which shows the proportion of different attenuation coefficients in the total detection results. 

**Figure 5:**
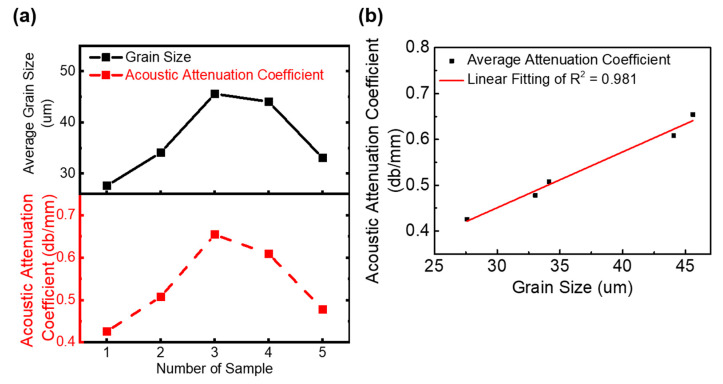
Comparison of characterization of grain size in Samples 1–5 between LOUD and EBSD. (**a**) The average grain size of Samples 1–5 was measured using EBSD (solid black line), and the acoustic attenuation coefficient of Samples 1–5 was detected using LOUD (dotted red line). (**b**) Linear fitting for grain size dependent on acoustic attenuation coefficient.

Figure 5a shows the measurements of the average grain size for Samples 1–5 by EBSD and LOUD detection, respectively. The measurements from Samples 1–3 show that the average grain size increased while the heat input increased. The measurements from Samples 3–5 showed that the average grain size decreased while the interlayer wait time decreased. The results showed that the decreasing heat input and interlayer wait time could refine grain size under the VP CMT arc mode [28]. As shown in Figure 5b, the measured acoustic attenuation coefficient was used to establish a linear relationship with the average grain size. The coefficient of determination of linear fitting (R^2^) was 0.981, which showed a good fit. 

### 3.2. Simultaneous Detection of Grain Size and Elements Using LOUD

**Figure 6:**
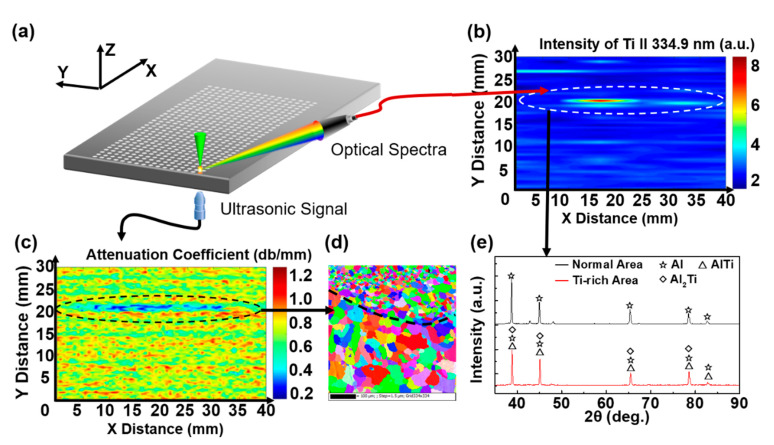
The measurements of Sample 3. (**a**) Schematic of LOUD mapping for Sample 3. (**b**) The mapping of Ti for Sample 3. (**c**) The mapping of the acoustic attenuation coefficient for Sample 3. (**d**) The EBSD map from the area of the abnormal acoustic attenuation coefficient (black dotted line area in (c)) in Sample 3. (**e**) The X-ray diffraction (XRD) pattern of normal areas and Ti-rich areas (white dotted line area in (**b**)) for Al and Al-Ti in Sample 3.

In order to investigate both the distribution of grain size and the elements in the WAAM sample, a large-area, high-precision mapping was performed on Sample 3 using LOUD detection. As shown in Figure 6a, the mapping area was 30 × 40 mm^2^ with a 0.5 mm step. The Ti mapping is shown in Figure 6b. Due to the layer-by-layer processing of WAAM, the distribution of Ti in the whole sample appeared to have a layer structured distribution, which was the same with the layers [4], especially the 20 mm position in the vertical direction (marked by a white dotted line). As shown in Figure 6c, the distribution of the acoustic attenuation coefficient, which could characterize the grain size distribution, was detected simultaneously. There was a layer structured distribution area with an abnormal reduction in the acoustic attenuation coefficient at the 20 mm position in the vertical direction (marked by a black dotted line), which was the same as the Ti-rich areas. As shown in Figure 6d, an EBSD map was obtained from the acoustic attenuation coefficient abnormal area. A distinct boundary of grain size distribution can be observed (marked by a black dotted line). The X-ray diffraction (XRD) pattern is shown in Figure 6e. The Al peak was the main spectral peak in the normal area. However, the spectral peaks were moved gradually from Al to AlTi and Al_2_Ti forms in the Ti-rich areas due to the crystallization of Ti and Al. The results indicated that the Ti had a refining effect on the grain size of the Al alloy, which was in good agreement with the results of recent studies [2,29]. The results of XRD verified the accuracy of the Ti distribution detected by LOUD. The LOUD results showed that the addition and enrichment of Ti elements would lead to the grain refinement of the Al alloy, which would affect the mechanical properties of the components. 

## 4. Conclusions

In conclusion, LOUD detection was developed to simultaneously detect the grain size and elements in AM components. The results showed that the grain size measurements of five WAAM Al alloy samples with different parameters were consistent with the results of EBSD, with a linear fitting of R^2^ 0.981. The Ti detection using LOUD showed Ti enrichment, which was verified by a moving XRD. The simultaneous detection of the distribution of grain size and Ti of an Al alloy WAAM sample by LOUD showed that the layer structured distribution of a Ti-rich area and grain size refinement area were obtained in the same area. The LOUD results indicated that Ti enrichment led to the abnormal crystallization of Ti and Al during manufacturing and finally resulted in grain size refinement. In addition, LOUD is time saving and nondestructive and shows great potential for studying the relationship between the alloy elements and mechanical properties of AM components. 

## Figures and Tables

**Table 1:** Processing Parameters.

Sample	Arc Current (A)	Arc Voltage (V)	Wire Feed (m/min)	Scanning Speed (mm/s)	Interlayer Wait-Time (s)	Heat Input (J/mm)
1	117	12	9	8.42	180	150
2	117	12	9	7.22	180	175
3	117	12	9	6.32	180	200
4	117	12	9	6.32	120	200
5	117	12	9	6.32	60	200

**Table 2:** Chemical compositions.

Elements (wt. %)	Cu	Mg	Si	Fe	Cr	Zr	Zn	Mn	Ti	Al
Wire (ER2319)	6.010	0.004	0.044	0.172	0.003	0.100	0.007	0.270	0.104	rest

## Abbreviations

AM	additive manufacture
LOUD detection	laser opto-ultrasonic dual detection
WAAM	wire + arc additive manufacturing
EBSD	electron backscatter diffraction
XRD	X-ray diffraction
CCD	charge-coupled device
SBR	signal-to- background ratio
DAQ	data acquisition card
DDG	digital delay generator
VP CMT	variable polarity cold metal transfer
